# Metabolomics-Based Analysis of Dayezhong Fresh Tea Leaves: Effects of Cultivar and Tenderness on Black Tea Quality

**DOI:** 10.3390/foods15142465

**Published:** 2026-07-12

**Authors:** Yibo Hu, Fei Ren, Wenxue Chen, Weijun Chen, Qiuping Zhong, Ming Zhang, Jianfei Pei, Ying Lyu, Haiming Chen, Wubin Wen, Liang Xu, Rongrong He

**Affiliations:** 1 HNU-HSF Collaborative Innovation Laboratory, College of Food Sciences & Engineering, Hainan University, 58 People Road, Haikou 570228, China; hyb23hndx@163.com (Y.H.); renfei0006@163.com (F.R.); hnchwx@vip.163.com (W.C.); chenwj@hainanu.edu.cn (W.C.); 990511@hainanu.edu.cn (Q.Z.); peijianfei@hainanu.edu.cn (J.P.); lyuying@hainanu.edu.cn (Y.L.); hmchen168@126.com (H.C.); 2 Haikou Key Laboratory of Special Foods, Haikou 570228, China; 3 Hainan State Farms Tropical Products Industry Group Co., Ltd., Haikou 570226, China

**Keywords:** Hainan Dayezhong, Yunnan Dayezhong, non-targeted metabolomics, fresh tea, black tea

## Abstract

To elucidate differences in fresh-leaf quality associated with cultivar and leaf tenderness at harvest, this study investigated three tea samples: Hainan Dayezhong with one bud and one leaf (1L-HN), Hainan Dayezhong with one bud and two leaves (2L-HN), and Yunnan Dayezhong with one bud and one leaf (1L-YN). Basic physicochemical indices, non-targeted metabolomics, and sensory evaluation were integrated for comprehensive analysis. The results showed that 1L-HN exhibited excellent sensory quality and contained abundant polyphenols and amino acids, thereby providing a material basis for its balanced taste profile. Metabolites such as L-tryptophan, 13(S)-HPOT, and linoleic acid were closely associated with the characteristic floral and fruity flavor of Hainan Dayezhong. Metabolomic analysis further indicated that flavonoid biosynthesis and glycerophospholipid metabolism were the major pathways contributing to the observed metabolic differences. This study provides a theoretical basis for understanding the mechanisms underlying flavor formation in different varieties of black tea.

## 1. Introduction

Tea is among the most widely consumed aromatic beverages worldwide. Among the six major types of tea, black tea accounts for approximately 75% of total global tea consumption and production [1]. Black tea is rich in tea polyphenols, which exhibit excellent antioxidant capacity and may help slow the aging process and reduce the risk of chronic diseases. Meanwhile, its flavonoids are beneficial for lowering cholesterol levels and improving cardiovascular health, while catechins can enhance immunity. Keemun black tea, Zhengshan Xiaozhong, and Jin Junmei are renowned for their elegant aroma and mellow flavor [2], and are mainly produced in Fujian and Anhui provinces. In contrast, Yunnan Dayezhong black tea, Hainan Dayezhong, and Yingde black tea are distinguished by their rich aroma and robust flavor [3], and are usually produced in southern China, including Yunnan, Hainan, and Guangdong provinces. Hainan Dayezhong is a highly representative tea germplasm resource in tropical China and has attracted considerable attention due to its unique geographical growing environment.

Hainan Dayezhong mainly grows on Hainan Island, the southernmost island province of China, which is characterized by a tropical climate with abundant sunshine, high temperatures, and high humidity throughout the year. This unique climate promotes the vigorous growth of tea plants and contributes to the accumulation of abundant chemical constituents, especially polyphenols and amino acids [4]. Black tea made from Hainan Dayezhong is usually characterized by a sweet fruity aroma and flavor, accompanied by a long-lasting aftertaste. In addition, the mineral-rich soil of Hainan further contributes to the layered flavor profile of the tea [5]. Carbon fixation and flavonoid biosynthesis have been reported as the main metabolic pathways involved in the processing of Hainan Dayezhong black tea, its chemical composition has been reported to be associated with the flavor quality of Hainan Dayezhong black tea [6]. Tea plants grown in Hainan Province share some morphological similarities with Yunnan Dayezhong; however, Hainan Dayezhong, which is characterized by buds lacking trichomes, represents a unique tea germplasm resource in Hainan [5]. To better distinguish Hainan Dayezhong tea from other varieties, previous studies have conducted gene sequencing analyses. The results showed that Hainan Dayezhong is genetically distinct from other tea germplasm resources and suggested that it should be identified as a new tea species, named *Camellia hainanensis* Sheng. Although gene sequencing has helped resolve the classification controversy, the differential compounds and key metabolic pathways that determine the quality of Hainan Dayezhong tea leaves remain to be elucidated. Meanwhile, the chemical composition of fresh leaves is crucial for the quality of subsequently processed tea. Therefore, the chemical differences in fresh leaves should be further investigated to reveal the unique quality characteristics of Hainan Dayezhong.

The processing of black tea and the tenderness of fresh leaves at harvest are important factors determining tea quality. Currently, the effects of processing steps, including withering, shaking, fermentation, and drying, on black tea quality have been reported [6,7]. However, the evidence has shown that Pan Long Gong Ou black tea (PCT) made from young leaves has a distinct sweet and fruity flavor, whereas tea made from mature leaves exhibits sour, grassy, and earthy notes, despite being processed using the same techniques [8]. Another study also showed that young leaves are more conducive to flavor enhancement, not only imparting a fresher taste to tea infusion but also increasing aroma intensity. In contrast, older leaves contain fewer aromatic substances and exhibit stronger bitterness and astringency [9]. It is worth noting that the sensory differences in finished black tea are closely related to the chemical constituents of its fresh leaves. However, no systematic study has investigated the chemical differences in Hainan Dayezhong fresh leaves with different tenderness levels or their correlation with the quality of finished tea.

Previous work has investigated metabolic variation during the processing of Hainan Dayezhong black tea and identified metabolites related to flavor development [6]. However, that study mainly focused on dynamic changes during black tea manufacturing. In contrast, this study focused on fresh tea leaves before processing and compared the effects of cultivar origin and leaf tenderness on metabolite profiles and sensory-related quality differences. By integrating basic physicochemical indices, non-targeted metabolomics, and sensory evaluation, this study aimed to characterize the metabolite composition of Hainan Dayezhong and screen potential metabolite candidates associated with its characteristic floral and fruity aroma.

## 2. Materials and Methods

### 2.1. Chemicals

Standard Rutin (≥99%), (−)−Epicatechin (EC), (−)−Epicatechin gallate (ECG), (−)−Epicatechin (EGC), (−)−Epicatechin gallate (EGCG), (−)−Catechin (C) (≥98% purity) were purchased from McLean Chemical Company (Shanghai, China). Methanol, acetonitrile and formic acid of chromatographic grade was obtained from McLean Chemical Company (Shanghai, China).

### 2.2. Tea Samples

The tea samples were collected on March 2024 in the Baima area of Hainan province. The three sample types were Hainan Dayezhong one bud one-leaf (1L-HN), one bud two-leaves (2L-HN), and Yunnan Dayezhong one bud one-leaf (1L-YN). Six independent biological replicates were collected for each sample type to ensure sample representativeness. Each biological replicate was harvested from distinct tea plants and handled separately throughout all subsequent experiments. The fresh tea leaves were picked and immediately frozen in liquid nitrogen to minimize field heat and metabolic changes. The frozen samples were then transported to Hainan University and stored at −80 °C for subsequent analysis.

### 2.3. Determination of Tea Polyphenol

The quantification of tea polyphenol content was based on the Chinese national standard GB/T 8313-2018 [10] with minor modifications. Tea samples (2 g) were added to methanol (50 mL) and then were immediately placed in a 70 °C water bath for 10 min with stirring every 5 min. Afterward, the mixture was filtered to collect the supernatant and the remaining residue was again added with 70% methanol for secondary extraction. Subsequently, the two combined supernatants (20 μL) were taken and reacted with 10% folinol reagent (100 μL) for 7 min before adding 7.5% sodium carbonate (80 μL). The content of tea polyphenols was analyzed using a microplate reader (Synergy LX, Biotek Instruments Inc., Winooski, VT, USA) at 765 nm when placed under light shielding for 1 h, with gallic acid as a reference.

### 2.4. Determination of Flavonoid

The secondary extract (50 μL) obtained in Section 2.3 was reacted with sodium nitrite solution for 5 min before adding aluminum nitrate (15 μL). NaOH (100 μL) was added as treatment and reacted for 15 min in the dark after continuing the reaction for 8 min. Absorbance was recorded at 510 nm using a microplate reader, and flavonoid content was expressed in terms of rutin equivalents [11].

### 2.5. Determination of Amino Acid

The determination of amino acids in black tea was carried out by using the protocol of China National Standard GB/T8314-2013 [12]. Briefly, 3.0 g of freeze-dried tea powder was extracted with 500 mL boiling water for 30 min and diluted to volume. An aliquot (1 mL) was reacted with phosphate buffer and ninhydrin, heated in a boiling water bath for 15 min, diluted to 25 mL, and the absorbance was measured at 570 nm. A calibration curve was prepared using theanine as the standard, and total free amino acids were expressed as theanine equivalents.

### 2.6. Determination of Catechins

The content of catechins was determined using high performance liquid chromatography (HPLC) (1260 infinity IISanta Clara, CA, USA) equipped with a C_18_ column according to the method (GB/T 8313-2018 [10]). Firstly, a series of gradient concentrations of EC, ECG, EGC, EGCG, C standard sample (solutions were prepared and then analyzed using the following conditions: Mobile phase A was 0.9% acetonitrile and 0.2% acetic acid with EDTA-2Na as stabilizer while mobile phase B was 80% acetonitrile and 2% acetic acid with EDTA-2Na as stabilizer. The flow rate was 1 mL/min and the column temperature was 35 °C with DAD value of 278 nm. A linear gradient elution and procedure was used as follows: at 0–10 min, 0% B; 10–15 min, 0–32% B; 15–25 min, 32% B; 25–30 min, 0% B. The samples were quantitatively analyzed by making a standard curve based on the peak area of the standard.

### 2.7. Non-Targeted Metabolomics Analysis

Metabolites in tea samples were extracted based on previous report method [13]. Briefly, tea samples were vacuum freeze-dried and then ground using a grinder (MM 400, Retsch, Haan, Germany) at 30 Hz for 1.5 min. Subsequently, powdered samples (50 mg) were mixed with pre-cooled 70% methanol solution (1200 μL). Intermittent extraction was performed using a vortex oscillator (VORTEX-5, Kylin-Bell, Nantong, China), with vortexing for 30 s every 30 min for a total of six cycles. After centrifugation at 13,400 × *g* for 3 min using a centrifuge (5424R, Eppendorf, Hamburg, Germany), the supernatant was filtered through a 0.22 μm microporous membrane for UPLC-MS analysis using a UPLC system (LC-30A, Shimadzu, Tokyo, Japan) coupled to a TripleTOF 6600+ mass spectrometer (SCIEX, Foster City, CA, USA).

The conditions of UPLC-MS/MS were performed on a Waters ACQUITY Premier HSS T3 column (1.8 µm, 2.1 mm × 100 mm), with 0.1% formic acid aqueous solution was used as mobile phase A, and 0.1% formic acid in acetonitrile was used as mobile phase B. The separation was carried out at a flow rate of 0.4 mL/min and the injection volume was 4 μL with the column temperature of 40 °C. The steps of gradient elution were as follows: 0–2 min, 5–20% B; 2–5 min, 20–60% B; 5–6 min, 60–99% B; 6–7.5 min, 99% B; 7.5–7.6 min, 99–5% B; 7.6–10 min, 5% B. Mass spectral full-scan data were in ESI (−) and ESI (+) modes with an acquisition time of 10 min, respectively. Spray ionization source temperatures were positive and negative at 550 °C and 450 °C. Ion spray voltages were 5000 v in positive ion mode and 4000 v in negative ion mode; Ion Source Gas1 was set to 50 psi; Ion Source Gas2 was set to 60 psi, and Curtain Gas was set to 35 psi. Quality control samples (QCs) were prepared by pooling aliquots of all sample extracts and were inserted regularly during instrumental analysis to assess analytical repeatability. Peaks with a missing rate greater than 50% in each sample group were filtered out, and missing values were imputed. After peak correction, metabolite features were putatively annotated by searching an in-house database, integrated public databases, predicted databases, and metDNA based on MS and MS/MS information. Annotation confidence was evaluated using a composite identification score based on fragment ion matching and spectral-library matching. Features with an identification composite score ≥ 0.5, MS1 mass error ≤ 25 ppm, MS/MS mass error ≤ 50 ppm, RT deviation ≤ 6 s, detection frequency ≥ 80% in biological replicates, and QC CV ≤ 30% were retained. The positive and negative ion modes were then merged, retaining the annotation with the highest confidence level and the lowest CV.

### 2.8. Sensory Evaluation

Sensory evaluation was conducted by a panel of six professionally trained tea tasters (three males and three females, aged 20–60 years), following previously reported sensory evaluation methods [14,15]. Sensory evaluation studies involving human participants conducted at Hainan University do not require approval from an ethics committee. All panelists voluntarily participated in the study and signed written informed consent forms before the evaluation. They were informed that they could withdraw from the study at any time, and no personal information would be disclosed without their explicit consent. This study did not involve vulnerable populations, such as minors, individuals with disabilities, or socially disadvantaged groups. Prior to the formal evaluation, all panelists received four training sessions over a two-week period to standardize the sensory descriptors used for black tea quality assessment. Tea infusion was prepared according to the national standard GB/T 23776-2018 [16], Methods for Sensory Evaluation of Tea . Briefly, 3.00 g of tea sample was infused with 150 mL of boiling water for 5 min and then filtered into a tasting bowl. The sensory quality of black tea was assessed based on five attributes: dry tea appearance, liquor color, aroma, taste, and infused leaf condition. Each attribute was scored on a 100-point scale and weighted by its corresponding coefficient, and the final sensory score was calculated as the sum of all weighted scores. Each sample was evaluated three times by each panelist, with an interval of approximately 60 s between evaluations, and the final results were expressed as mean values.

### 2.9. Data Analysis

The experiment was repeated three times for each analysis and the results were expressed as mean ± standard deviation (SD). The data of the experiment were analyzed by one-way ANOVA using SPSS software (IBM 27.0, Armonk, NY, USA), where *p* < 0.05 was considered a significant difference. The obtained data were plotted using Origin 2021 (Origin Lab Co., Northampton, MA, USA) in this study.

## 3. Results and Discussion

### 3.1. Sensory Evaluation Results

In this study, three groups of tea samples (1L-HN, 2L-HN, and 1L-YN) were subjected to sensory evaluation and scored based on five attributes: appearance, liquor color, aroma, taste, and infused leaves (Table S1). Significant differences in sensory attributes were observed among the three groups. The 1L-HN sample obtained the highest overall score (91.76), with a tightly twisted appearance, rich aroma characterized by distinctive floral and fruity notes (93.17), fresh and mellow taste with a sweet aftertaste, bright orange-yellow liquor color, and soft, bright infused leaves. The 1L-YN sample exhibited a rich and persistent aroma accompanied by a light nutty note (90.33). Its taste was mellow, and the liquor color showed a deep reddish-brown hue (91.83). The infused leaves were slightly thick but relatively uniform. The aroma and taste of the 2L-HN sample were slightly weaker (88.83), with slight astringency, a relatively coarse appearance, and lower uniformity of infused leaves, although its liquor color remained bright. These results indicate that the three tea samples had distinct sensory characteristics. Overall, 1L-HN achieved the highest comprehensive sensory score, suggesting that black tea made from 1L-HN had the best sensory quality among the tested samples.

### 3.2. Content of Major Compounds in Tea Samples

**Figure 1:**
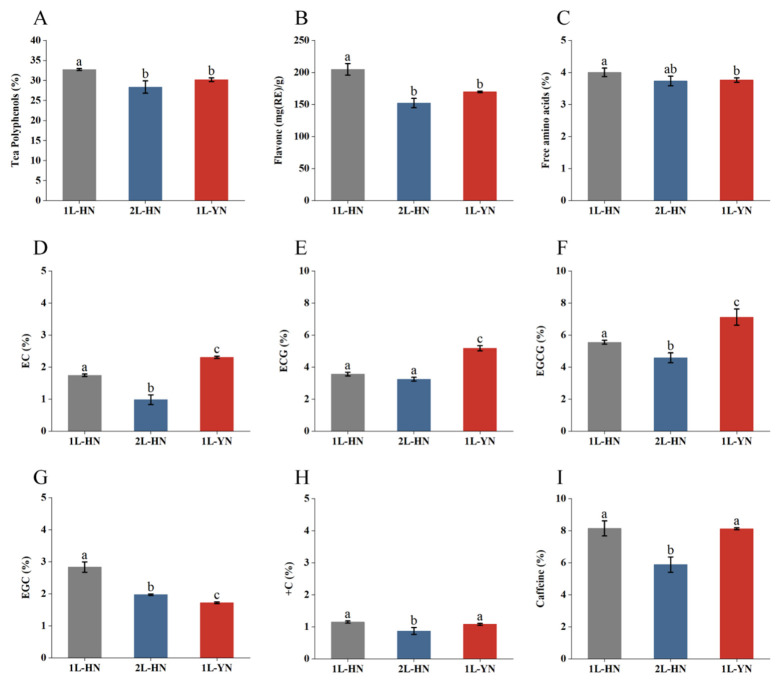
Comparison of the major biochemical components among the 1L-HN, 2L-HN, and 1L-YN samples: (**A**) tea polyphenol content; (**B**) flavone content; (**C**) free amino acid content; (**D**) epicatechin (EC) content; (**E**) epicatechin gallate (ECG) content; (**F**) epigallocatechin gallate (EGCG) content; (**G**) epigallocatechin (EGC) content; (**H**) catechin [(+)−C] content; and (**I**) caffeine content. Values are presented as mean ± standard deviation. Regarding the determination of the basic indexes of tea, black represents 1L-HN, blue represents 1L-YN, and red represents 1L-2N in the figure. *p* < 0.05 was used to do the analysis of variance, and a, b, c are the analysis of variance within groups.

The tea polyphenol content was determined, and the results are shown in Figure 1A. The tea polyphenol content accounted for approximately 25–35% of the dry weight of fresh tea leaves, which is consistent with previous findings [17]. Among the three samples tested, 1L-HN tea has the highest tea polyphenol content (32.7%). The tea polyphenol content of 1L-YN and 2L-HN did not differ much at 30.18% and 28.35%, respectively. The flavonoid content exhibited significant variations across samples, where 1L-HN demonstrated the highest concentration of 205 mg (RE)/g, significantly exceeding 1L-YN at 169.66 mg (RE)/g and 2L-HN at 152.23 mg (RE)/g (Figure 1B). It has been revealed that flavonoids are important contributors to the aroma and flavor of black tea [18]. The higher flavonoid content in the 1L-HN sample likely contributes to the multidimensional richness of the processed black tea’s aroma profile richness and enhanced palate mellowness. A previous study found that the amino acid content in tea was about 3–4%, while playing an important role in the refreshment of tea. The amino acid content of 1L-HN (4%) was higher than that of 1L-YN (3.73%), while 2L-HN (3.76%) was not significantly different from either.

Catechins are divided into ester catechins and non-ester catechins, of which ester catechins include ECG, EGCG0 and non-ester catechins include C, EC, EGC, etc. Ester catechins have strong astringent properties and usually give tea a strong astringent taste; non-ester catechins are refreshing and slightly astringent, which play an important role in the softness of the taste of tea broth [19]. Compared with 1L-HN and 2L-HN, 1L-YN showed higher contents of EC, ECG, and EGCG (Figure 1C–F), accounting for 2.31%, 6.84%, and 7.12%, respectively. These catechins are important precursors for the subsequent formation of thearubigins, which may partly explain the reddish liquor color of 1L-YN. The higher contents of gallated catechins may also be related to the stronger taste intensity of 1L-YN. Although most catechins in 1L-HN were lower than those in 1L-YN, the EGC content in 1L-HN was much higher than that in the other two samples (Figure 1G). Gallated catechins, such as ECG and EGCG, are generally considered important contributors to the bitterness and astringency of tea infusion. Therefore, the higher contents of ECG and EGCG in 1L-YN may be associated with its stronger and more astringent taste [20]. In contrast, the relatively higher EGC content in 1L-HN may reflect differences in catechin composition and may be related to the mellow and refreshing taste characteristics of this sample. Caffeine is mainly associated with bitterness in tea infusion. As shown in Figure 1I, the caffeine contents of 1L-HN and 1L-YN were corrected to 4.89% and 4.87%, respectively, both of which were significantly higher than that of 2L-HN (4.12%). These results suggest that higher caffeine contents may contribute to stronger bitterness and taste intensity.

### 3.3. Overview of Metabolomics Results

**Figure 2:**
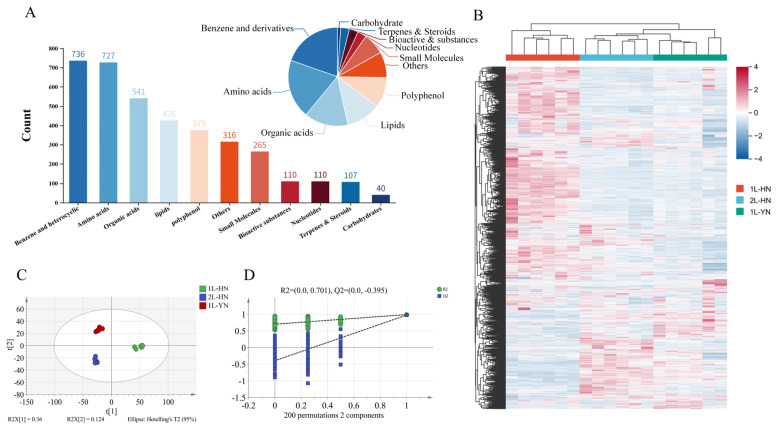
On the overall characteristics of the three teas. (**A**) Histograms and pie charts of the classification of the substances detected by metabolomics, the height of the histograms represents the content of the detected substances in the classification. (**B**) Heat map showing the three teas, each column represents a different tea variety, each group has 6 samples, red represents up-regulation and blue represents down−regulation. (**C**,**D**) OPLS−DA and 200 permutation tests.

The samples were analyzed using UPLC-MS/MS to obtain comprehensive metabolite profiles across tea samples differing in cultivar and leaf tenderness. The overlay analysis of total ion chromatograms (TICs) from the QC samples (Figure S1) showed good reproducibility of metabolite extraction and instrumental analysis, indicating the high stability of the analytical procedure. In total, 3753 putatively annotated metabolite features were detected across the samples, including 2345 in positive ion mode and 1408 in negative ion mode, of which 2345 were in the positive ion mode and 1408 were in the negative ion mode; mainly including 736 benzene and heterocyclic compounds, 736 amino acids and its derivatives, and 541 organic acids (Figure 2A).

As shown in Figure 2B, the clustered heatmap illustrated the overall relative abundance patterns of metabolites among the samples. Hierarchical clustering showed that samples from the same group generally exhibited similar metabolite abundance patterns, while differences were observed among 1L-HN, 1L-YN, and 2L-HN. These results suggest that both cultivar origin and leaf tenderness may contribute to variations in the metabolite profiles of Dayezhong tea fresh leaves. Considering the large number of detected metabolite features, the heatmap was mainly used to show the overall clustering trend rather than to interpret individual metabolite changes.

The identified metabolites were further subjected to orthogonal projections to latent structures-discriminant analysis (OPLS-DA) to remove variations unrelated to group discrimination. After filtering out non-correlated variation by OPLS-DA (Figure 2C), the model showed good explanatory and predictive performance, with R^2^X = 0.56, R^2^Y = 0.985, and Q^2^ = 0.935. Clear separation was observed among the three sample groups, indicating significant differences in their metabolite profiles. Overall, 1L-HN, 1L-YN, and 2L-HN were clearly separated from each other, while samples within each group clustered closely, indicating good experimental repeatability and reliable data quality. Notably, 1L-YN and 2L-HN showed no obvious separation along the first component but were clearly separated along the second component. In the OPLS-DA permutation test (Figure 2D), the intercept of the Q^2^ regression line was below zero. As the degree of permutation increased, the Q^2^ values of the permuted models gradually decreased, indicating that the original model had good robustness and no obvious overfitting.

### 3.4. Differential Metabolites (DMs)

**Figure 3:**
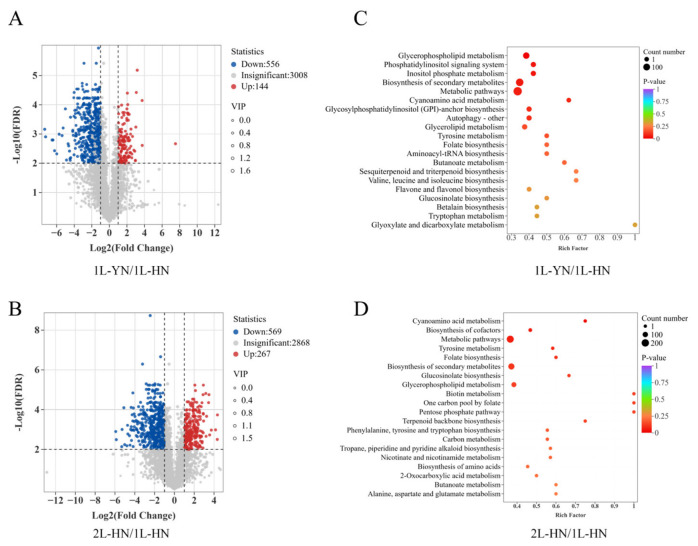
Differential metabolites (DMs) were differentially analyzed for 1L-YN VS 1L-HN and 2L-HN vs. 1L-HN using the criteria VIP ≥ 1 and FC ≥ 2 or ≤0.5. (**A**) Volcano plots of differential metabolites 1L-YN vs. 1L-HN; (**B**) volcano plots of differential metabolites 2L-HN VS 1L-HN; (**C**) KEGG pathway enrichment analysis of differential metabolites in 1L-YN vs. 1L-HN. The horizontal axis represents the Rich Factor of each pathway, the vertical axis represents the enriched KEGG pathways, the color scale indicates the *p*-value, and the point size represents the number of differential metabolites enriched in each pathway; (**D**) KEGG pathway enrichment analysis of differential metabolites in 2L-HN vs. 1L-HN.

To further compare the metabolite profiles among samples differing in cultivar and leaf tenderness, differential metabolites (DMs) were screened using a combination of multivariate and univariate statistical criteria, with 1L-HN used as the reference group. VIP values were obtained from the OPLS-DA model, and fold change (FC) was calculated based on relative metabolite abundance. Raw *p*-values were calculated using Student’s *t*-test and then adjusted for multiple testing using the Benjamini–Hochberg false discovery rate (FDR) method. Metabolites with VIP > 1, FC ≥ 2 or ≤0.5, and FDR-adjusted *p*-value < 0.05 were considered significant DMs. The results were visualized using volcano plots (Figure 3A,B). Compared with 1L-HN, 1L-YN showed 144 up-regulated and 556 down-regulated metabolites, whereas 2L-HN exhibited 267 up-regulated and 569 down-regulated metabolites. The larger number of down-regulated metabolites in both comparison groups indicated that many differential metabolites were more abundant in 1L-HN, which was consistent with the clustering heatmap. Metabolites and metabolic pathways closely associated with black tea sensory quality were further screened and summarized in Table 1 and Table 2.

### 3.5. Important Differential Metabolites (DMs)

#### 3.5.1. Amino Acid

Amino acids have been reported to be important contributors to the fresh taste and flavor of tea [21]. Therefore, amino acid composition was further analyzed in this study (Table 1). A total of 28 free amino acids were identified in fresh tea leaves, including theanine, aspartic acid, and proline, some of which are also related to aroma formation.

Previous studies have shown that theanine is the most abundant free amino acid in fresh tea leaves, accounting for 54.5–57.4% of total free amino acids [22]. Theanine is mainly synthesized in tea roots and transported to tea buds and young leaves through the plant metabolic system, where it participates in nitrogen metabolism and transport in tea plants [23].

Comparison between 1L-HN and 1L-YN revealed significant differences in amino acid composition. The contents of L-tryptophan and asparagine in 1L-HN were significantly higher than those in 1L-YN (Table 1), whereas no significant difference in theanine content was observed between the two groups. L-Tryptophan is involved in plant growth and the regulation of photosynthesis, while asparagine is closely associated with the fresh taste and overall taste quality of tea. These results suggest that the differential accumulation of these amino acids may play an important role in the formation of quality differences between the two samples.

To investigate the influence of leaf tenderness on amino acid composition, the amino acid profiles of 1L-HN and 2L-HN were compared, and the results are presented in Table 2. The contents of L-phenylalanine and L-glutamic acid increased as the tenderness of Hainan Dayezhong leaves decreased. A previous study showed that glutamic acid and phenylalanine can reach their highest levels in tea leaves with one bud and two leaves [24]. As an important fresh-tasting amino acid, L-glutamic acid contributes to the fresh taste of tea infusion. Therefore, differences in fresh-tasting amino acids may partly explain the sensory differences between samples with different tenderness levels. Moreover, phenylalanine, as a precursor of aromatic compounds, may provide possible metabolic clues related to aroma differences among the samples [25], thereby providing a possible explanation for the differences in aroma characteristics among the samples. However, this potential role was inferred based on previous reports and the metabolomic differences observed in this study, and requires further validation by direct flavor chemistry experiments.

#### 3.5.2. Flavonoid

As important secondary metabolites in plants, flavonoids and flavonoid glycosides play multiple roles in plant growth and development. Therefore, flavonoids and flavonoid glycosides in fresh leaves of Hainan Dayezhong and Yunnan Dayezhong were further analyzed. A total of 333 flavonoid-related compounds were detected, among which 58 showed significant differences. According to their glycoside types, these compounds can be classified into flavonol-C-glycosides (FCGs) and flavonol-O-glycosides (FOGs). FOGs can be further divided into kaempferol-O-glycosides (KOGs), myricetin-O-glycosides (MOGs), and quercetin-O-glycosides (QOGs) [26]. Flavonoids such as kaempferol-3-O-(2″-p-coumaroyl) galactoside, kaempferol 3,4′,7-triacetate, and quercetin 3-O-(6″-acetyl-glucoside) were significantly more abundant in 1L-HN than in 1L-YN. In contrast, quercetin-3-O-(2″-p-coumaroyl) galactoside and myricitrin were more abundant in Yunnan Dayezhong. Flavonoid glycosides, mainly in the form of O-glycosides, have been reported as low-threshold bitter compounds in tea infusion [27]. During black tea fermentation and processing, flavonoid glycosides may undergo enzymatic transformation, which can reduce bitterness and astringency. Meanwhile, β-glucosidase, a hydrolytic enzyme involved in tea processing, can catalyze the hydrolysis of flavonoid glycosides, thereby enhancing the aromatic complexity and layered flavor profile of tea [28]. In this study, 2,5-dimethyl-4-hydroxy-3(2H)-furanone beta-D-glucopyranoside and benzyl beta-D-glucopyranoside were more abundant in 1L-HN. Both compounds belong to the beta-glucopyranoside class and may serve as important glycosidically bound aroma precursors during black tea processing [29]. Therefore, their higher abundance in 1L-HN may partly contribute to the richer aroma characteristics of black tea made from this sample.

In the comparison of samples with different tenderness levels, 1L-HN showed higher levels of several flavonoid compounds and derivatives, including quercetin, kaempferol, and cyanidin-related compounds. In this study, flavonol galactosides such as kaempferol-3-O-(6″-malonyl) galactoside and quercetin-3-O-(6″-malonyl) galactoside gradually decreased with decreasing leaf tenderness. Quercetin-3-O-(2″-O-galactosyl) glucoside, pelargonidin 3-O-rutinoside, and phloretin-4′-O-(6″-feruloyl) glucoside showed similar trends to the above flavonol galactosides (Table 2). In contrast, apigenin-8-C-glucoside was more abundant in mature leaves, which is consistent with previous studies [30]. In addition, quercetin-3-O-(2″-p-coumaroyl) galactoside, apigenin-7-O-gentiobioside, and quercetin-3-O-(2″-galloyl) arabinogalactoside were up-regulated in 2L-HN. These differences in flavonoid composition may provide possible metabolite clues for understanding the sensory variations among tea samples with different tenderness levels.

### 3.6. Key Differential Metabolic Pathways

**Figure 4:**
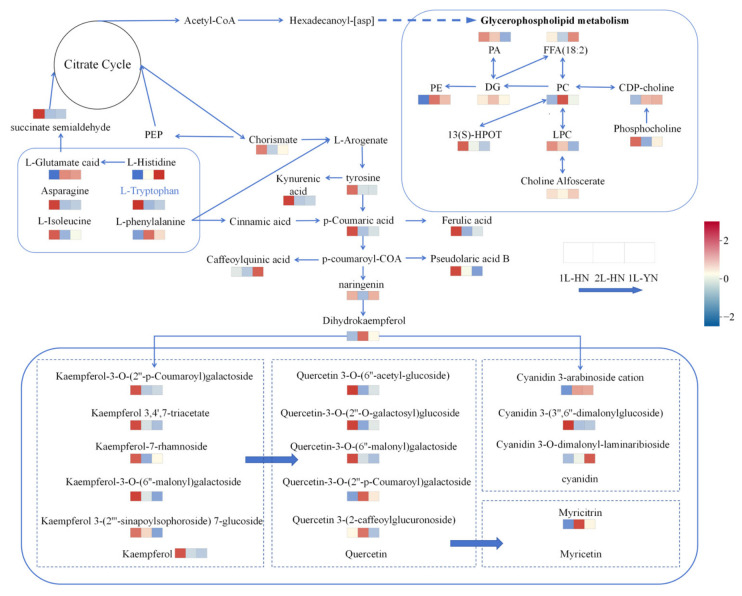
Comparison of the pathways of formation of key compounds with VIP > 1 in the three teas, with solid arrows representing direct effects and dashed arrows representing indirect effects on metabolite pathways. Red and blue colors represent up-regulation of substances and down-regulation of substances, and yellow color represent no significant changes. Boxes represent from left to right 1L-HN, 1L-YN,2L-HN.

Pathway analysis of the identified DMs was performed to reveal the key biochemical pathways associated with fresh leaves of different cultivars and tenderness levels, and the results are shown in Figure 3C,D. KEGG enrichment analysis showed that glycerophosphate metabolism, phenylalanine, tyrosine and tryptophan metabolism, and terpene metabolism were the main pathways contributing to the differences between 1L-HN and the other two samples, 1L-YN and 2L-HN. In addition, flavonoid metabolism, citrate cycle (TCA cycle), and glycerophospholipid metabolism were further examined based on the KEGG database. A proposed metabolic network was constructed by integrating pathway information with the metabolite profiles of fresh tea leaves from different cultivars and tenderness levels (Figure 4). In the KEGG enrichment analysis, pathways with higher Rich Factor values and lower *p*-values indicated a greater concentration of differential metabolites. The enriched pathways were mainly related to amino acid metabolism, flavonoid metabolism, glycerophospholipid metabolism, and the TCA cycle. These pathways are biologically relevant to tea quality because amino acids are associated with fresh taste and aroma precursor formation, flavonoids are related to bitterness, astringency, color, and antioxidant properties, and lipid-related pathways may provide precursors for aroma-related compounds. Therefore, these enriched pathways provide possible metabolic clues for understanding the differences in sensory quality among tea samples. The relative abundance of DMs among samples was further visualized using heatmaps.

The tricarboxylic acid cycle (TCA cycle) plays a pivotal role in plant growth and metabolism by providing energy and important carbon skeletons for the biosynthesis of amino acids, lipids, and other metabolites [31]. Amino acid biosynthesis is usually initiated from phosphoenolpyruvate (PEP), which has been reported to increase significantly in black tea after the withering stage [32]. As seen by the quantitative analysis of metabolic pathway maps, it was found by quantitative metabolomics analysis that among the key node amino acids in the metabolic network of flavonoid synthesis and the tricarboxylic acid cycle (TCA cycle), L-phenylalanine, L-glutamic acid and L-histidine showed a significant up-regulation trend in the 1L-YN and 2L-HN samples It should be noted that the relative amounts of the main amino acids mentioned above were significantly lower in the 1L-HN samples, presumably due to their further conversion into metabolites such as succinate semialdehyde and p-Coumaric acid, which are involved in energy metabolism or the synthesis of secondary metabolites. Meanwhile, some amino acids that did not directly enter this metabolic pathway, such as Asparagine, L-Tryptophan and L-Isoleucine, were relatively high in 1L-HN, which might play a role in nitrogen storage or maintenance of total amino acid levels during metabolic regulation.

Glycerophospholipid metabolism was prominently enriched in the KEGG pathway analysis. As important components of plant cell membranes, phosphatidylcholines (PCs) and phosphatidic acids (PAs) not only contribute to membrane structure but are also involved in the response of tea plants to abiotic stress [26]. They also have an impact on the taste of black tea, for example, the fatty acids released during black tea processing are partially derived from PCs. Therefore, it has been suggested that the fatty acid content of black tea can be used to determine the degree of fermentation of black tea [33]. The PCs content of 2L-HN was significantly up-regulated from the quantitative thermogram. Lipids can be interconverted, PCs in 1L-HN can be converted to 13(S)-hydroperoxynicotinic acid (13(s)-HPOT), and PCs in 1L-YN to linoleic acid (FFA18:2). We can see that FFA (18:2) was higher in 1L-YN than the other two, and FFA (18:2) is one of the main precursors of C6 volatiles. During continued mechanical damage, such as during black tea rolling, linoleic acid may promote the formation of jasmonolactone, and these volatiles give black tea a fresh and green grassy flavor [34]. This may be the reason for the difference in aroma between Hainan and Yunnan tea broths. Phosphatidic acids (PAs) improved the resistance of tea leaves. It is also an important aromatic precursor substance that affects the aroma composition of tea [26]. As lipid-related metabolites involved in stress responses and aroma formation, PAs may participate in lipid-derived aroma precursor pathways.

Flavonoid metabolism is one of the major secondary metabolic pathways in tea and plays an important role in tea flavor and health-related properties. Figure 4 illustrates the relationship between the major flavonoids and their transformations. L-phenylalanine is a precursor substance for flavonoid metabolism and is subsequently converted to coumaroyl A and trans-coumaroyl coenzyme A. Light can induce the accumulation of flavonoids, especially in bright light, and flavonoids protect tea cells by reducing the production of reactive oxygen species (ROS) accumulation [35]. The higher contents of kaempferol and quercetin in 1L-HN than in 1L-YN may be due to the greater accumulation of 1L-YN into cyanidin in the flavonoid metabolic pathway.

Although the expression of flavonoid biosynthesis-related enzymes was not examined in this study, previous studies have shown that key enzymes such as phenylalanine ammonia-lyase (PAL), flavonol synthase (FLS), and chalcone synthase (CHS) exhibit relatively high expression levels in tea shoots and leaves [30]. Therefore, we hypothesize that the activity or expression levels of these key enzymes may differ between 1L-HN and 2L-HN, thereby affecting flavonoid accumulation. This may partly explain why the flavonoid content in 1L-HN was significantly higher than that in 2L-HN. This young leaf-specific accumulation pattern may be associated with the developmental regulation of tea plants, as young tissues generally require higher levels of flavonoids to respond to environmental stress.

Kaempferol and quercetin derivatives are relatively stable compounds and have been reported as important metabolic markers for distinguishing different tea plant varieties [36]. As shown in Figure 4, most kaempferol and quercetin derivatives, such as kaempferol 3,4′,7-triacetate, kaempferol-7-rhamnoside, and quercetin 3-O-(6″-acetyl-glucoside), showed higher abundances in the 1L-HN group. In contrast, cyanidin derivatives, such as cyanidin 3-arabinoside cation and cyanidin 3-O-dimalonyl-laminaribioside, were more abundant in the 1L-YN group. Therefore, these differentially accumulated flavonoids may serve as potential markers for distinguishing Hainan Dayezhong tea from Yunnan Dayezhong tea.

Overall, these pathway-level results suggest that the metabolic differences among the tea samples were not determined by single metabolites alone, but were associated with coordinated changes in amino acid metabolism, lipid metabolism, and flavonoid metabolism. For varietal differences, the higher levels of some amino acids, lipid-related metabolites, and kaempferol/quercetin derivatives in 1L-HN may provide possible metabolic clues for its sensory characteristics. For tenderness differences, changes in phenylalanine-related metabolism and flavonoid accumulation may be related to the differences between 1L-HN and 2L-HN. Therefore, the integrated metabolic network provides a broader explanation for the potential relationship between fresh-leaf metabolism and tea quality differences.

### 3.7. DMs Correlation Analysis

We screened the substances in the DMs and major metabolic pathways mentioned in the above text. Metabolites previously reported to contribute to the taste or aroma of tea infusion were selected and categorized, and Pearson correlation coefficients among these metabolites were calculated. Their relationships were visualized using a correlation heatmap (Figure S2).

The correlation analysis revealed relationships among differential metabolites associated with taste and aroma attributes. These metabolites were mainly divided into two groups according to their potential contributions to tea sensory quality. The first group was mainly associated with taste characteristics of tea infusion, including amino acids and polyphenols. Among them, fresh-tasting amino acids, such as L-glutamic acid, showed negative correlations with certain phenolic acids, suggesting a possible association between the accumulation of bitter or astringent compounds and changes in fresh taste. L-Glutamic acid also showed negative correlations with several other metabolites, such as L-tryptophan (r = −0.86) and folic acid (r = −0.89), indicating that interactions among these metabolites may influence the overall flavor balance of tea. The second group of metabolites was mainly associated with floral and fruity aroma characteristics, including asparagine, 13(S)-HPOT, and D-phenylalanine. The strong correlations among these metabolites suggest that they may be jointly involved in the formation of aroma characteristics in tea. These metabolites were more abundant in 1L-HN than in 1L-YN and 2L-HN, suggesting that the accumulation of these amino acids and lipid oxidation-related metabolites may contribute to the enhanced floral and fruity aroma of 1L-HN. This is consistent with the richer aroma and higher aroma sensory score observed for 1L-HN. This study further analyzed the correlations between metabolite abundance and sensory scores. The results showed that aroma was the sensory attribute most closely associated with metabolite variation. It showed strong positive correlations with L-tryptophan (r = 0.93, r = 0.87), asparagine (r = 0.90, r = 0.87), D-phenylalanine (r = 0.91, r = 0.83), and 4-hydroxycinnamic acid (r = 0.84, r = 0.80), suggesting that these metabolites may be closely related to the formation of floral and fruity aroma characteristics in black tea. Taste scores were also positively correlated with L-tryptophan (r = 0.70, r = 0.73) and L-isoleucine (r = 0.68, r = 0.70), although the correlations were relatively weaker, indicating that these amino acids may make certain positive contributions to favorable taste quality. In contrast, vitexin showed clear negative correlations with both aroma and taste scores, with correlation coefficients of r = −0.73 for aroma and r = −0.70, r = −0.75 for taste, suggesting that vitexin may be associated with lower aroma and taste scores. Overall, these results indicate that differences in black tea sensory quality may be associated with coordinated changes in specific amino acids, phenolic acids, and flavonoids. Overall, the correlation analysis provides useful clues for understanding the metabolite basis of sensory differences among tea samples from different origins and tenderness levels.

## 4. Conclusions

The results showed that Hainan Dayezhong fresh leaves contained higher levels of tea polyphenols and flavonoids and were rich in characteristic metabolites, such as β-glucopyranosides, FFA 18:2, and 13(S)-HPOT, which may be potentially associated with the unique floral and fruity aroma characteristics of Hainan Dayezhong black tea. The abundances of kaempferol and quercetin derivatives were higher in Hainan Dayezhong than in Yunnan Dayezhong, whereas cyanidin derivatives showed higher abundance in Yunnan Dayezhong. These structurally stable flavonoids may serve as potential markers for distinguishing Hainan Dayezhong from Yunnan Dayezhong. In addition, key metabolic pathways, including phenylalanine metabolism and glycerophospholipid metabolism, were associated with differences in flavor-related metabolite profiles. Because metabolomics analysis was performed on fresh leaves, these associations should be further validated using processed tea samples and targeted flavor chemistry or functional studies. This study provides a theoretical basis for optimal raw material selection and offers new insights into the potential relationship between fresh-leaf metabolites and quality formation in black tea.

## Figures and Tables

**Table 1:** Differential metabolites of 1L-YN and 1L-HN were screened according to FC ≥ 2 or FC ≤ 0.5, VIP > 1, and FDR < 0.05. Some of the substances screened are shown in the table below. Class indicates the classification of the substance. FDR indicates false discovery rate after validation of multiple hypothesis testing. Type indicates the type of metabolites that were up-regulated or down-regulated.

Compounds	Class	VIP	FDR	Fold Change	Log2FC	Type
L-Glutamic acid	Amino acid and Its derivatives	1.32	<0.01	2.16	1.11	up
L-Isoleucine	Amino acids and Its derivatives	1.03	0.04	0.41	−1.28	down
L-Tryptophan	Amino acids and Its derivatives	1.60	<0.01	0.43	−1.23	down
Asparagine	Amino acids and Its derivatives	1.52	<0.01	0.14	−2.81	down
D-phenylalanine	Amino acids and Its derivatives	1.55	<0.01	0.38	−1.38	down
Kaempferol 3,4′,7-triacetate	Flavonoids	1.04	<0.01	0.32	−1.64	down
Kaempferol-3-O-(2″-p-Coumaroyl) galactoside	Flavonoids	1.21	0.02	0.09	−3.44	down
Quercetin 3-O-(6″-acetyl-glucoside)	Flavonoids	1.53	<0.01	0.39	−1.37	down
Quercetin-3-O-(2″-p-Coumaroyl)galactoside	Flavonoids	1.11	0.01	5.62	2.49	up
Kaempferol 3-(2′′′-sinapoylsophoroside) 7-glucoside	Flavonoids	1.11	0.03	0.23	−2.13	down
Kaempferol-7-rhamnoside	Flavonoids	1.13	0.04	0.35	−1.51	down
Kaempferol-3-O-(6″-malonyl)galactoside	Flavonoids	1.48	<0.01	0.20	−2.33	down
Kaempferol	Flavonoids	1.32	0.01	0.44	−1.71	down
Vitexin	Flavonoids	1.47	<0.01	2.69	1.43	down
Cyanidin 3-(3″,6″-dimalonylglucoside)	Flavonoids	1.51	<0.01	0.06	−4.11	down
Cyanidin 3-O-dimalonyl-laminaribioside	Flavonoids	1.30	0.02	3.96	1.99	up
Benzyl beta-d-glucopyranoside	Benzene and substituted derivatives	1.39	<0.01	0.45	−1.17	down
2,5-dimethyl-4-hydroxy-3(2H)-furanone beta-D-glucopyranoside	Others	1.03	0.01	0.29	−1.79	down
Folic acid	Organic acid and Its derivatives	1.46	0.01	0.49	−1.04	down
Homovanillic acid	Organic acid and Its derivatives	1.45	0.01	0.43	−1.23	down
4-Hydroxycinnamic acid	Phenolic acids	1.28	0.01	0.30	−1.72	down
4-O-Methylgallic acid	Phenolic acids	1.60	<0.01	0.27	−1.90	down
Salvianolic acid L	Phenolic acids	1.23	0.01	0.19	−2.43	down
Melitric acid A	Phenolic acids	1.13	0.01	0.47	−1.10	down
Kynurenic acid	Amino acid and Its metabolites	1.46	<0.01	0.36	−1.46	down

**Table 2:** Differential metabolites of 2L-HN and 1L-HN were screened according to FC ≥ 2 or FC ≤ 0.5, VIP > 1, and FDR < 0.05. Some of the substances screened are shown in the table below. Class indicates the classification of the substance. FDR indicates false discovery rate after validation of multiple hypothesis testing. Type indicates the type of metabolites that were up-regulated or down-regulated.

Compounds	Class	VIP	FDR	Fold_Change	Log2FC	Type
L-Phenylalanine	Amino acids and Its metabolites	1.24	0.01	2.55	1.35	up
L-Glutamic acid	Amino acids and Its metabolites	1.32	<0.01	2.16	1.11	up
Kaempferol-3-O-(6″-malonyl)galactoside	Flavonoids	1.48	<0.01	0.36	−1.45	down
Pelargonidin 3-O-rutinoside	Flavonoids	1.22	<0.01	0.48	−1.05	down
Phloretin-4’-O-(6″-Feruloyl)glucoside	Flavonoids	1.48	<0.01	0.08	−3.59	down
Vitexin	Flavonoids	1.43	0.01	3.37	1.75	up
Quercetin-3-O-(2″-p-Coumaroyl)galactoside	Flavonoids	1.17	0.01	12.03	3.59	up
apigenin-7-O-gentiobioside	Flavonoids	1.45	<0.01	2.35	1.23	up
Quercetin-3-O-(2″-Galloyl)Arabinoside	Flavonoids	1.13	0.05	2.46	1.30	up
Kaempferol 3,4’,7-triacetate	Flavonoids	1.32	<0.01	0.41	−1.28	down
Kaempferol-7-rhamnoside	Flavonoids	1.40	<0.01	0.05	−4.33	down
Quercetin 3-O-(6″-acetyl-glucoside)	Flavonoids	1.43	<0.01	0.26	−1.97	down
Quercetin-3-O-(6″-malonyl)galactoside	Flavonoids	1.52	<0.01	0.18	−2.44	down
Quercetin-3-O-(2″-O-galactosyl)glucoside	Flavonoids	1.48	<0.01	0.05	−4.36	down
Quercetin-3-O-(2′′′-Caffeoyl)sophoroside	Flavonoids	1.35	<0.01	3.17	1.66	up
Cyanidin 3-arabinoside cation	Flavonoids	1.15	0.04	2.15	1.11	up
Cyanidin 3-(3″,6″-dimalonylglucoside)	Flavonoids	1.43	<0.01	0.03	−5.28	down
Folic acid	Organic acid and Its derivatives	1.38	0.01	0.42	−1.26	down
Homovanillic acid	Organic acid and Its derivatives	1.39	<0.01	0.32	−1.63	down
Ferulic acid	Phenolic acids	1.44	<0.01	0.46	−1.10	down
4-Hydroxycinnamic acid	Phenolic acids	1.27	<0.01	0.22	−2.16	down
4-O-Methylgallic acid	Phenolic acids	1.52	<0.01	0.15	−2.71	down
Melitric acid A	Phenolic acids	1.35	<0.01	0.34	−1.56	down
Kynurenic acid	Amino acid and Its metabolites	1.38	<0.01	0.33	−1.58	down

## Data Availability

The original contributions presented in this study are included in the article/Supplementary Materials. Further inquiries can be directed to the corresponding author.

## Supplementary Materials

The following supporting information can be downloaded at: https://www.mdpi.com/article/10.3390/foods15142465/s1, Figure S1. Overlay of the total ion flow map (TIC plot) for mass spectrometric detection of QC samples. A is in positive ion mode and B is in negative ion mode. Figure S2. Differential metabolites with taste and aroma were screened out and correlations between them were calculated. 1L-HN was used as a reference to compare 1L-YN and 2L-HN. The My way metabolic analysis package (R version 4.1.2) was used, with the function package (psych 2.2.9), with basic analysis parameters: use = ‘pairwise’, method = ‘pearson’, adjust = ‘none’, alpha = 0.05. Table S1. Sensory evaluation was conducted according to five aspects: Dried tea, aroma, taste, soup colour and leaf base. The score for each indicator is 100 points, taking the average of the scores of the six reviewers, and the final overall score is calculated by weighting: 25% for dry tea, 10% for soup colour, 25% for aroma, 30% for taste, and 10% for leaf base, and taking one decimal place.
