# Supplementary material for: Metabolomics-Based Analysis of Dayezhong Fresh Tea Leaves: Effects of Cultivar and Tenderness on Black Tea Quality
Source: Foods. 2026 Jul 12;15(14):2465. doi: 10.3390/foods15142465 (PMC13408195; doi:10.3390/foods15142465)
Supplement: Supplementary file 1 [file foods-15-02465-s001.zip › foods-4391104-supplementary.pdf]

Figure S1 Overlay of the total ion flow map (TIC plot) for mass spectrometric detection of QC samples.A is in positive ion mode and B is in negative ion mode.

A

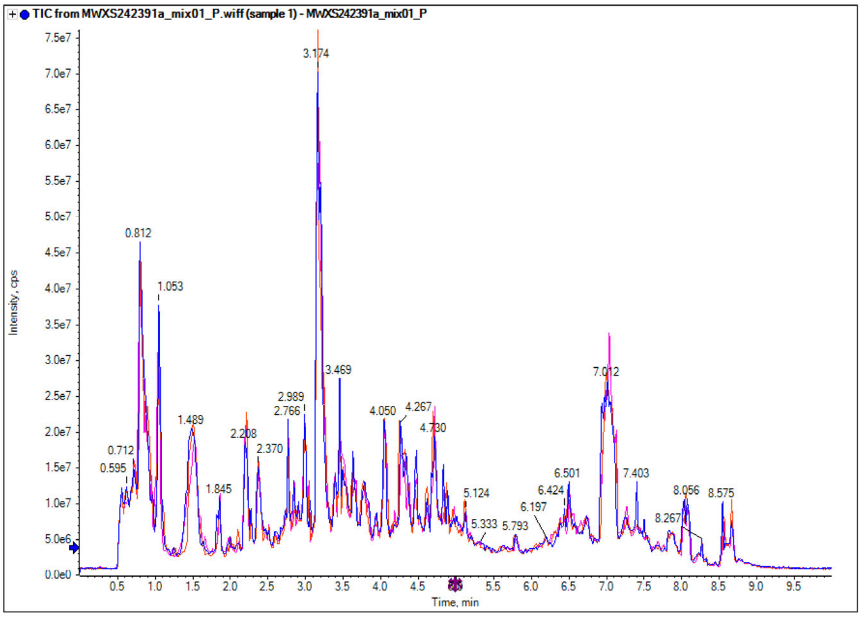

7/14/2024 9:34:17 AM

B

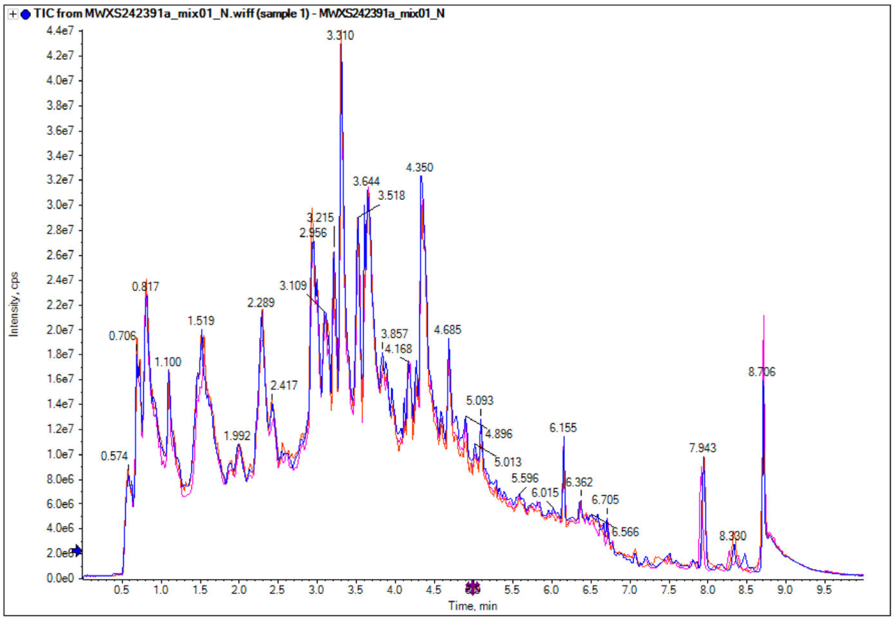

7/18/2024 1:47:30 PM

Figure S2 Differential metabolites with taste and aroma were screened out and correlations between them were calculated. 1L-HN was used as a reference to compare 1L-YN and 2L-HN. The My way metabolic analysis package (R version 4.1.2) was used, with the function package (psych 2.2.9), with basic analysis parameters: use=‘pairwise’, method=‘pearson’, adjust=‘none’, alpha=0.05.

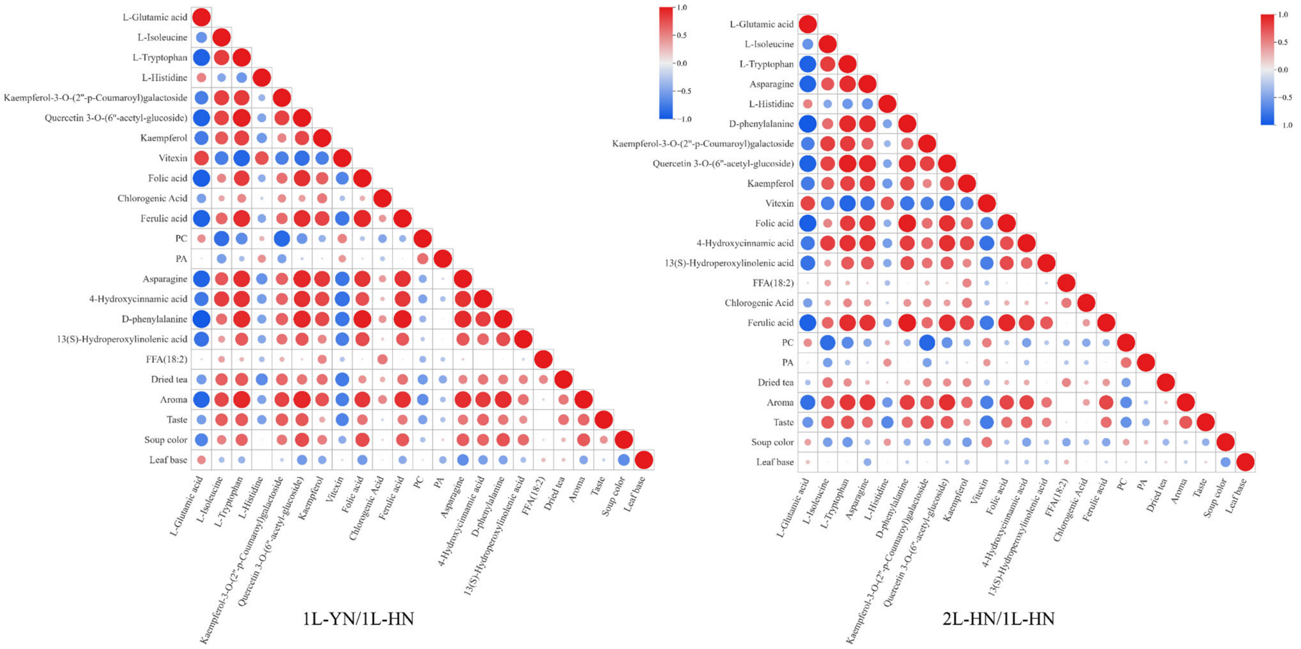

Table S1. Sensory evaluation was conducted according to five aspects: Dried tea, aroma, taste, soup colour and leaf base. The score for each indicator is 100 points, taking the average of the scores of the six reviewers, and the final overall score is calculated by weighting: 25% for dry tea, 10% for soup colour, 25% for aroma, 30% for taste, and 10% for leaf base, and taking one decimal place.

Sample / Evaluation indicators	1L-HN	1L-YN	2L-HN
Dried(100)	moist color, slightly golden hairs(91.33±1.21[a])	moist color with brown,slightly thick texture(90.00±1.41[ab])	moist color, less hairs showing(88.83±1.47[b])
Aroma (100)	distinctive fruity and floral aroma, long honeyed aroma(93.17±0.75[a])	rich fruity aroma, nutty aroma(90.33±1.03[b])	soft sweetness, slightly fruity aroma(88.67±1.03[c])
Taste (100)	fresh and sweet, slight sweetness(91.67±1.63[a])	rich and sweet flavor, slightly nutty flavor(89.00±0.89[b])	soft in the mouth, sweet with astringency(89.00±1.41[b])
Soup color (100)	bright orange-yellow(90.50±1.05[a])	reddish-brown, deep and thick(91.83±1.17[a])	bright orange-red, good light transmission(88.33±1.63[b])
Leaf base (100)	soft and lively, orange-yellow color(90.83±1.47[a])	slightly thicker leaves, reddish-bright color(91.00±0.89[a])	heavier leaves, slightly lower uniformity(91.83±1.67[a])
Composite indicators	91.76±0.72[a]	90.07±0.26[b]	89.09±0.53[c]
